# Effects of Jianpi Lishi Jiedu granules on colorectal adenoma patients after endoscopic treatment: study protocol for a randomized, double-blinded, placebo-controlled clinical trial

**DOI:** 10.1186/s13063-022-06236-6

**Published:** 2022-04-23

**Authors:** Hao Wu, Yuzhen Huang, Lu Yang, Kunhan Su, Shuo Tian, Xin Chen, Siyu Li, Wanli Liu

**Affiliations:** 1 Nanjing University of Chinese Medicine, Nanjing, China; 2 Nanjing Integrated Traditional Chinese and Western Medicine Hospital Affiliated with Nanjing University of Chinese Medicine, Nanjing, China

**Keywords:** Jianpi Lishi Jiedu granules, Colorectal adenomas, Recurrence, Clinical efficacy, Randomized controlled trial, Study protocol

## Abstract

**Background:**

Colorectal adenomas (CRAs) are precancerous lesions of the large intestine presenting as colorectal polyps. At present, the conventional treatment methods for CRA mainly include high-frequency electrocoagulation and electroexcision, biopsy forceps polypectomy, cauterization by laser and microwave, and other endoscopic interventions. The principal advantages conferred by these treatment strategies include less trauma, quick postoperative recovery, and simplicity to perform. However, the higher recurrence rates and insignificant improvement of postoperative symptoms after endoscopic surgery are considerable drawbacks to this approach. Besides, there is currently no effective pharmacotherapy to prevent the recurrence of CRA. Jianpi Lishi Jiedu (JLJ) granules are a form of traditional Chinese medicine (TCM) used to manage postoperative patients with CRA, which has shown a certain degree of efficacy in clinical practice. However, its effectiveness and safety profile have not been convincingly evaluated. The purpose of this study is to evaluate the clinical efficacy and safety profile of JLJ granules in the management of postoperative patients with CRA and to observe the recurrence rate of adenoma in these patients.

**Methods:**

A randomized, double-blind, and placebo-controlled clinical trial is performed in this study. A total of 80 postoperative patients with CRA will be randomly classified into the Jianpi Lishi Jiedu granules group or the placebo control group. Patients in both groups shall receive 3 months of intervention, after which medical follow-up and safety evaluation will be performed for all of the patients. The primary outcome is the recurrence rate of adenomas within 12 months. The secondary outcomes are the cardinal TCM symptom scores, minor TCM symptom scores, Bristol Stool Scale, efficacy of TCM symptoms, safety indicators, and blinding assessment.

**Discussion:**

In this study, the impact on the recurrence of adenomas and the efficacy and safety of JLJ granules in terms of improving the clinical symptoms of postoperative patients with CRA will be evaluated.

**Trial registration:**

Trial registration Chinese Clinical Trial Registry ChiCTR 2100044297. Registered on March 16, 2021

**Supplementary Information:**

The online version contains supplementary material available at 10.1186/s13063-022-06236-6.

## Background

Colorectal adenomas (CRAs) are benign epithelial protruding lesions originating from the colorectal mucosa or submucosa, projecting into the intestinal lumen [1]. CRAs are colorectal polyps, which have several pathological subtypes including adenomatous polyps, hyperplastic polyps, inflammatory polyps, and hamartomatous polyps. All of which belong to precancerous lesions in the epithelial tumor classification [2]. Among them, adenomatous polyps have the highest incidence and the highest potential for malignant transformation. The “Inflammatory polyp-adenoma-adenocarcinoma” sequence has become a recognized classical canceration pathway for colorectal cancer (CRC). As per statistics, about 95% of CRC originate from the canceration of adenomatous polyps [3]. With the advancement of endoscopic and pathological diagnostic techniques, the detection rate of colorectal polyps has been increasing year after year, especially with respect to adenomas [4]. Early intervention and blocking the “adenoma-adenocarcinoma” sequence can significantly reduce the risk of canceration and improve the quality of life in these patients. The mechanism of onset, recurrence, and canceration of CRA is not completely clear. Some scholars believe that the pathogenesis and recurrence of CRA might be multifactorial (due to factors such as heredity, immunity, the environment, etc.) [5, 6]. Currently, the conventional treatment methods for CRA include high-frequency electrocoagulation and electroexcision, biopsy forceps polypectomy, cauterization by laser and microwave, and other endoscopic interventions [7]. Although these interventions generally entail less trauma incurred by the patients, quick postoperative recovery, and simplicity of operation, surgeons also face some clinical difficulties related to these interventions; for example, the symptoms experienced by patients cannot be completely improved after endoscopic resection, and microscopic resection cannot reduce the recurrence rate. Studies at home and abroad [8, 9] have indicated that the average recurrence rate of patients with CRA is 51.4% within 1 year after surgery; meanwhile, that of patients with progressive CRA is 59.5% within 1 year after surgery and as high as 78.07% within 5 years after surgery. Moreover, the feasibility of endoscopic resection is significantly limited in patients with diffusing colorectal polyps of different sizes.

Most patients with CRA would not display specific symptoms, and frequent colonoscopy screenings would cause a huge economic burden to patients. Besides, there is currently no reliable treatment plan or measure to prevent recurrence for post-surgical CRA patients. The focus of most clinical studies is non-steroidal anti-inflammatory drugs (NSAIDs), such as aspirin, sulindac, and celecoxib [10, 11]. There are also some studies on other drugs such as beneficial intestinal bacteria preparations [12–14], but their clinical application value remains debatable, and they have not been widely recognized and applied. Thus, clinicians and surgeons face the immense challenge of developing safe and effective drugs for symptomatic relief in postoperative CRA patients, as well as preventing recurrence and avoiding canceration.

Over the recent years, with the in-depth research carried on TCM, people are increasingly aware of its unique advantages in the prevention and treatment of CRA [15, 16]. Our team found that in clinical practice, Jianpi Lishi Jiedu granules developed from a classic Chinese herbal medicine formula can not only relieve the symptoms of patients with CRA after endoscopic treatment, but also reduce the recurrence rate of adenoma [17]. However, its exact clinical efficacy, mechanism of influence on the recurrence of adenomas, and safety profile have not been convincingly evaluated. Therefore, this protocol, a randomized, double-blinded, and placebo-controlled trial, has been designed in this study to further clarify the clinical efficacy of the Jianpi Lishi Jiedu recipe in patients with CRA after endoscopic surgery, observe the recurrence rate of adenomas, and evaluate its safety profile.

## Methods/design

### Study design

**Fig. 1:**
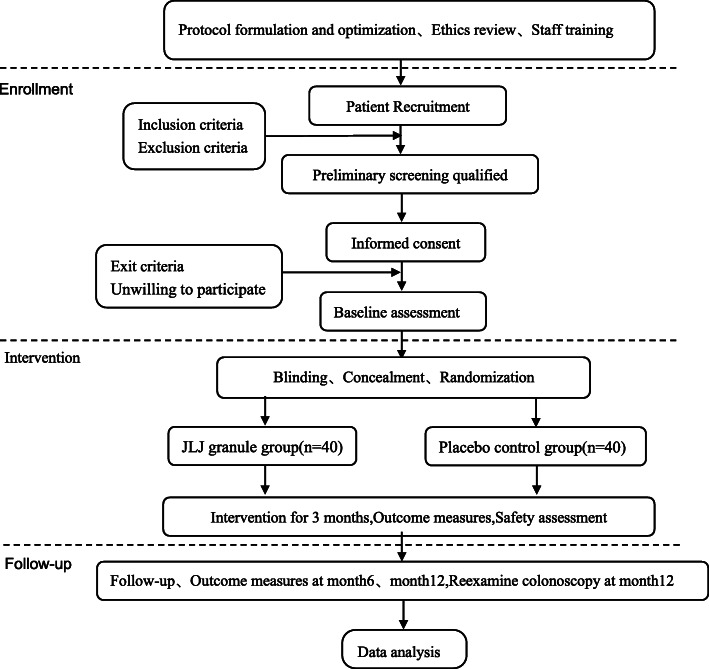
The flowchart of the protocol

**Fig. 2:**
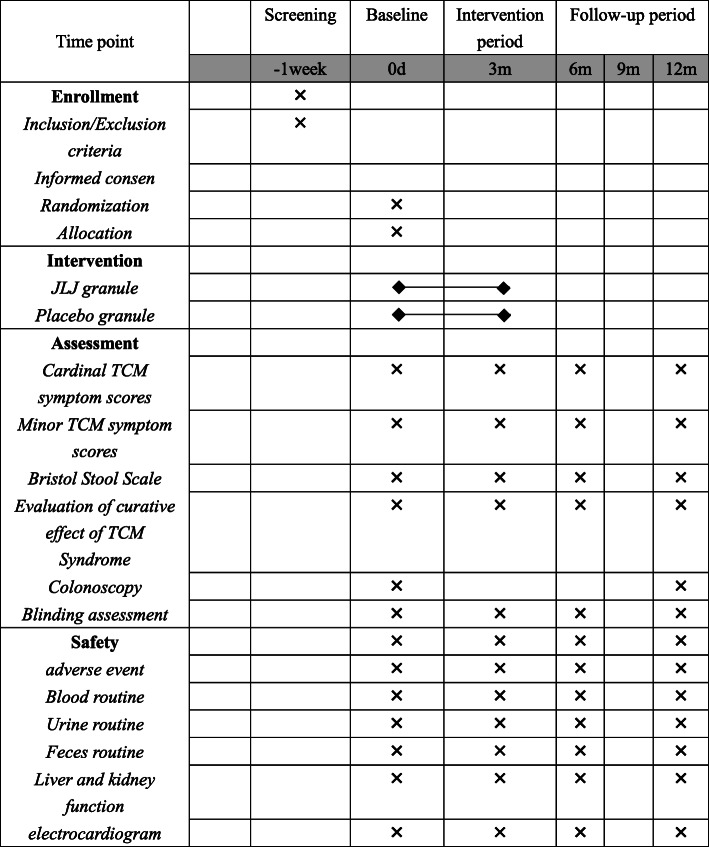
The proposed project schedule

This study presents a randomized, double-blinded, and placebo-controlled clinical trial performed in accordance with the Declaration of Helsinki, the Standard Protocol Items: Recommendations for Interventional Trials (SPIRIT) 2013 Statement [18], and the Standard Protocol Items for Clinical Trials with Traditional Chinese Medicine: Recommendations, Explanation and Elaboration (SPIRIT-TCM) Extension 2018 Statement [19] from June 2021 to June 2023. In this study, a total of 80 eligible patients will be enrolled and randomly classified into the JLJ granules group or the placebo control group. All the patients voluntarily will sign an informed consent before being included. Each included patient will be numbered in sequence and randomly classified into two groups using a ratio of 1:1. Participants in the JLJ granules group will be instructed to take JLJ granules (110 g per day) for 3 consecutive months, while participants in the placebo control group will be given placebo granules with the same appearance and smell as JLJ granules but no therapeutic effect (110 g per day) for 3 consecutive months. All of the researchers and patients will be blinded right from the beginning of the trial. All the clinical data will be recorded before the intervention (0 week) and after the intervention (at month 3, month 6, and month 12), and the results of the electronic colonoscopy will be recorded at month 12. The proposed project schedule is illustrated as a flowchart in Figs. 1 and 2.

### Objective and hypothesis

This study’s principal objective is to assess the recurrence of CRA, conduct an evaluation of the clinical efficacy of JLJ granules in the treatment of postoperative patients with CRA, and explore the safety profile of JLJ granules in the treatment of postoperative patients with CRA. In this study protocol, it will be assumed that, compared with the placebo, JLJ granules have better effects in terms of the recurrence rate of CRA, major symptom alleviation, the remission rate of minor symptoms, the Bristol stool scale, and the efficacy for patients with CRA after surgery, with no significant adverse effects despite an adequate safety profile.

### Participants

In this study, patients will be recruited in the outpatient department, Department of Gastroenterology and gastrointestinal endoscopy center of Nanjing Integrated Traditional Chinese And Western Medicine Hospital, and diagnosed according to the results of electronic colonoscopy and histopathology. All of the 80 patients included must voluntarily sign an informed consent. Data collection will be performed by two professionally trained researchers to ensure that the data is relatively objective and accurate.

### Inclusion criteria

1. Patients diagnosed with colorectal polyps through colonoscopy and adenoma through histopathological examination and received resection of the adenoma with biopsy clips or endoscopic resection within 4 weeks, including snare polypectomy (SS), endoscopic mucosal resection (EMR), piecemeal EMR (pEMR), endoscopic submucosal dissection (ESD), etc.
2. Patients with TCM syndromes of spleen-asthenia with excessive damp; refer to “Guidance principle of clinical study on new drug of traditional Chinese medicine” [20]. The clinical symptoms of spleen-asthenia with excessive damp are stated in Table 1. The cardinal symptoms in line with 2 items and the minor symptoms in line with 1 item shall be diagnosed as spleen-asthenia with excessive damp
3. Patients with age ranging from 18 to 65 years old, regardless of gender
4. Patients that voluntarily accepted observation and signed the informed consent and ethical principles

**Table 1:** The scores of TCM syndrome

Symptoms	Scores of TCM symptoms			
	None	Mild	Moderate	Severe
	0	1	2	3
**Cardinal TCM symptom**				
Diarrhea or mucus	None	Diarrhea or mucus, defecate 3–4 times/day	Diarrhea or mucus, defecate 5–10 times/day	Diarrhea or mucus, defecate>10 times/day
Constipation	None	Dry stool, defecate 1 time/day	Dry stool, defecate once/2–3 days	Very dry stool, defecate once/5–7 days
Abdominal pain	None	Occasionally, mild abdominal pain	Sometimes, moderate abdominal pain	Often, severe abdominal pain
Abdominal distension	None	Occasionally, mild abdominal distension	Sometimes, moderate abdominal distension	Often, severe abdominal distension
Inappetence	None	Mild, less than 1/3 reduction in food intake	Moderate, more than 1/3 reduction in food intake	Severe, no eating all day long
Dry or sticky feeling in the mouth	None	Mild, occasionally	Moderate, sometimes	Severe, often
**Minor TCM symptom**				
Fatigue	None	Mild, be able to insist on light physical work	Moderate, barely stick to daily activities	Severe, inactive all day
Bowel sound	None	Mild, occasionally	Moderate, sometimes	Severe, often
Nausea or vomiting	None	Mild, occasionally	Moderate, sometimes	Severe, often
Tongue and pulse:				

### Exclusion criteria

1. Patients not falling under the diagnostic criteria for colorectal polyps or with pathologies not considered within the diagnostic criteria for adenoma or TCM syndromes of spleen-asthenia with excessive damp
2. Pregnant or lactating women or those preparing to have children
3. Patients with an allergic constitution or an allergy to multiple drugs, drug addicts, and alcoholics
4. Patients with comorbidities such as serious cardiovascular, pulmonary, hepatic, renal, and hematologic diseases
5. Patients suspected of canceration or in whom canceration has been confirmed by biopsy
6. Patients taking drugs or undergoing other clinical trials within 1 month
7. Patients with severe physical disabilities (such as deafness, blindness, intellectual disability, mental disability, etc.) who are unable to cooperate or unwilling to cooperate

### Withdrawal criteria/dropout criteria/termination criteria

1. Patients not in line with the aforementioned inclusion criteria
2. Patients with incomplete clinical data, lost during medical follow-up, or requested to be withdrawn from the trial
3. Patients who requested to be withdrawn from the trial, patients with poor compliance or not following the drug administration protocol or with a change in the test drugs or administration of banned drugs
4. Patients with serious changes in the disease course or complications during the trial
5. Patients with serious adverse events during the trial, resulting in the inability to continue the trial

### Randomization

In this clinical trial, 80 postoperative patients with CRA will be randomly distributed into a JLJ granule (treatment) group or the placebo control group in a ratio of 1:1, according to the randomized sequence table generated by SAS 9.4 (SAS Institute Inc, Cary, NC, USA), with each patient attributed a number ranging from 01 to 80; each of these codes is unique to a specific patient. The test designer will decide the number series for the JLJ granule group and the control group. JLJ granules and placebo granules will be numbered and labeled with the same serial numbers as described above. An independent statistician from a third-party statistical agency, the trial designer, and the drug administrator will participate in the randomization, but will not be involved in the study process and the statistical analysis of the test results.

### Allocation concealment and blinding

The drug administrator will number the outer packages of drug packaging based on a random numerical sequence table. The independent statistician will prepare the allocation sequence table based on the above randomization and random numerical table, encrypt the computer software, and make 3 copies. Said copies will be kept by the independent statistician, the trial designer, and the drug administrator, respectively. The random numerical table, allocation sequence table, and numbers selected by relevant patients will be sealed in opaque envelopes. Before the trial completion, the group allocations will be masked, and all the patients, clinicians, nurses, data managers, statisticians, and other personnel will be blinded to the trial, and the random sequence table and number must be kept strictly confidential. The blinding can only be uncovered with the consent of the main researchers after completion of the trial and data analysis or in case an emergently severe adverse event occurs. Placebo and JLJ granules will be both packed in opaque packages, which will be exactly the same in appearance, color, smell, taste, dosage, and packaging. For emergency unblinding during the study, relevant cases will be considered as dropped out.

### Intervention

#### JLJ granules group

Patients randomly classified into the JLJ granules group will be provided with Jianpi Lishi Jiedu granules. Administration method: A pack of granules (110 g/pack) is dissolved into 150 ml of warm water every day and divided into 2 equal doses for administration at 2 separate times. The JLJ granules are produced by Tiangjiang Pharmaceutical Co., Ltd. (Jiangyin), with a herbal formula comprising 15 g of pilose asiabell roots, 10 g of fried *Atractylodes macrocephala*, 30 g of glabrous greenbrier rhizome, 15 g of *Forsythia suspensa*, 30 g of honeysuckle stem, and 10 g of *Cynanchum roseum*.

#### Placebo control group

Patients in the control group will be provided with placebo control granules. The administration method will be the same as with the JLJ granules. The dosage of each pack of placebo control granules must also be 110 g. The main components of the placebo control granules include starch, maltodextrin, caramel pigment, etc., free from any therapeutic effect.

### Study period

The course of intervention is set as 3 months. Follow-ups will be conducted at months 3, 6, and 12, and clinical data will be recorded again, and the reexamine of colonoscopy will be undergone at month 12. A safety evaluation will be carried out for all the patients after treatment initiation (comprising blood routine, urine routine, stool routine, liver and kidney function, and electrocardiography).

### Outcomes

#### Primary outcome

The primary outcome of this study is the recurrence rate of CRA within 12 months: refer to the Diagnostic Criteria and Therapeutic Effect for Diseases and Syndromes in Traditional Chinese Medicine (Chinese Medicine Industry Standards of the People's Republic of China; ZY/T001.1–94); recurrence refers to the formation of new adenomatous lesions after complete resection of the initial lesions, appearing at the original affected site or at other sites. The recurrence rate of adenomas = the number of patients with recurrence/the number of intra-group patients × 100%. The size, number, location, pathological type, etc. of CRAs will also be recorded.

#### Secondary outcome

1. Cardinal and minor TCM symptom scores: refer to “Guidance principle of clinical study on new drug of traditional Chinese medicine” [20]. The scores and details of TCM syndrome are provided in Table 1
2. Bristol Stool Scale [21]: type 1: the stool forms separate hard lumps and is difficult to pass; type 2: the stool is sausage-shaped, with a lumpy surface; type 3: the stool is strip-shaped, with cracks on the surface; type 4: the stool is strip-shaped, soft and smooth; type 5: the stool forms soft lumps; type 6: the stool is mushy; type 7: the stool is watery.
3. Safety indicators: including blood routine, urinalysis, stool routine, liver and kidney functions, and electrocardiography
4. The efficacy of TCM symptoms: refer to the Diagnostic Criteria and Therapeutic Effect for Diseases and Syndromes in Traditional Chinese Medicine (Chinese Medicine Industry Standards of the People’s Republic of China; ZY/T001.1–94)
5. Clinical control: After treatment, symptoms and signs almost disappeared and patients returned to normal activities and work. The efficacy rate for syndromes is ≥90%.
6. Excellent efficacy: After treatment, the signs and symptoms and the results of examinations improved significantly. The efficacy rate for syndromes is ≥60% but < 90%.
7. Efficacy: After treatment, improvement of the symptoms and signs and examination results was observed. The efficacy rate for syndromes is ≥30%, but < 60%.
8. Lack of efficacy: After treatment, symptoms and signs and the results of examinations did not improve compared to before the treatment. The efficacy rate for syndromes was < 30%.
9. Blinding assessment: To determine whether the blind is successful, all of the cases will be asked to guess which drug (JLJ granule, placebo granule, uncertain) they have received at months 0, 3, and 6 and month 12, and relevant results will be recorded by the statistician from the third-party statistical agency. If participants do not choose “uncertain,” the reason why they had made that assumption will also be recorded.

### Additional information

Moreover, general information (such as gender, age, and BMI) and disease-related information (such as patient’s family history, adenoma location, adenoma morphology, adenoma size, etc.) will be obtained while enrolling the patients and during follow-up.

### Adverse events

During the whole study process, a close observation must be conducted on the occurrence of adverse events. Any adverse events shall be recorded in the standard SAE form. Patients experiencing serious adverse events must be subjected to a close follow-up and managed appropriately. Detailed reports of every adverse event will be sent to the Ethics Committee of Nanjing Integrated Traditional Chinese And Western Medicine Hospital.

### Oversight and quality control

In order to ensure the accuracy and objectivity of this experiment, a third-party testing institution will be employed, and all of the data collection and quality control will be completed and recorded by trained investigators. Additionally, endoscopic resection of the adenomas must be a senior digestive endoscopic physician and supervised by another senior digestive endoscopic physician to ensure complete removal of the adenoma. Investigators from the third-party testing institution must be on the field to carry out the supervision and record the relevant data.

### Quality control of herbs

All the Chinese herbal medicine granules and control placebo granules to be used in this study are manufactured by Tiangjiang Pharmaceutical Co., Ltd. (Jiangyin, China), and all the Chinese herbal medicines and auxiliary materials meet the criteria in the *Good Agricultural Practice for Chinese Crude Drugs* of the National Medical Products Administration.

### Ethics and registration

This study protocol has been approved by the Ethics Committee of Nanjing Integrated Traditional Chinese And Western Medicine Hospital (No.202102, Additional files 1 and 2). All the patients voluntarily signed the informed consent (Additional files 3 and 4) before registration. This study has been registered with the Chinese Clinical Trial Registry (ChiCTR) (URL: http://www.chictr.org.cn/, No. ChiCTR2100044297).

### Sample size

The sample size is estimated to the recurrence rate of CRAs as the primary outcome of the trial; test level: *α* = 0.05, test efficacy: 1−*β*; 1−*β* = 0.9, so *β* = 0.1. Take *α* = 0.05 (bilateral), *β* = 0.1, *Z*_*α*/2_ = *Z*_0.05/2_ = 1.96, and *Z*_0.10_ = 1.282, the sample size of the treatment group and the control group each account for 50%, thus, Q1 = Q2 = 0.5. As per the previous literature [22], we assumed that the recurrence rate of adenomas of the TCM treatment group is about 20% (p1 ≈ 0.2), and the recurrence rate of the placebo control group is roughly 35% (p2 ≈ 0.35). We use the PASW(V.18.0) software; as per the two sample rate calculation formula: *n* = ((*Z*_*α*/2_√ (p(1−p)(Q1^−1^ + Q2^−1^)) + *Z*_*β*_√((p_1_(1−p_1_)/Q_1_) + (p_2_ (1−p_2_)/Q_2_))/(p_1_−p_2_))^2^, we can conclude that *n* ≈ 66. Taking into account patients’ withdrawal and loss during follow-up, we also assumed that the dropout rate in each group will be 15%, implying that *n* = 80, namely 40 cases in the treatment group and the control group, respectively.

### Statistical analysis

All data will be entered and counted by professional statisticians using SAS 9.4 (SAS Institute Inc., Cary, NC, USA) and SPSS 26.0 (IBM, Armonk, NY, USA). If the data is normally distributed and the variance is uniform, the measurement data will be expressed as mean ± standard deviation (*x* ± *s*). The paired *t*-test will be adopted for intra-group comparison before and after treatment, and the independent sample *t*-test will be adopted for inter-group comparison before and after treatment. If the data does not follow a normal distribution or has an uneven variance, the measurement data will be expressed as median (quartile interval) [M (Q)], and the Wilcoxon rank sum test will be employed for data analysis before and after treatment. The chi-square test will be adopted to compare the rates between both groups. *P*<0.05 means that the difference is statistically significant.

## Discussion

With the development of endoscopic technology and the improvement of people’s awareness, the detection rate of CRA has been rising over the past few years. In China, CRC is one of the most prevalent malignant tumors of the digestive system, only less prevalent than gastric cancer, liver cancer, and esophageal cancer [23]. CRA is one of the precancerous lesions leading to CRC. Previous studies [24, 25] suggest that it takes nearly 10 years for CRC to develop from early stage to advanced stage, and about 95% of CRC originate from malignant transformation of CRA. Therefore, the research focus of gastroenterologists has been the early detection of precancerous CRC lesions, inhibiting off the “adenoma-adenocarcinoma” pathway and preventing the occurrence of CRC. At present, the conventional treatment methods for CRA consist of endoscopic surgery, including endoscopic biopsy forceps removal, snare polypectomy (SS), endoscopic mucosal resection (EMR), piecemeal EMR (pEMR), endoscopic submucosal dissection (ESD), etc. [26]. All of them have such advantages as less trauma, quick postoperative recovery, and simplicity of operation. Nonetheless, some studies have reported [27] that patients with CRA still have a high recurrence rate after endoscopic surgery, and endoscopic interventions cannot solve the problem of adenoma recurrence. After endoscopic resection of CRA, the symptoms experienced by most patients cannot be relieved, including changes in stool properties or defecating habits, abdominal pain, and abdominal discomfort. Furthermore, there is currently no recognized and effective drug to prevent the recurrence of adenomas. Patients require regular follow-up and reexamination through colonoscopy. If recurrence occurs, endoscopic surgery is required again, which might lead to great emotional and financial burden for patients and society as a whole. Hence, how can we improve the efficacy in the treatment of colorectal polyps, prevent recurrence and canceration via drug intervention has become a priority in gastroenterology [28].

According to TCM principles, the occurrence and recurrence of CRA are related to the patient’s “constitution.” Due to the fact that the patient’s constitution does not change after endoscopic surgery, CRA can easily recur. Over our past 10 years of experience in clinical diagnosis and treatment, our team has concluded that CRA is related to the patient’s “spleen-asthenia” constitution according to principles of TCM, and “excessive damp accumulation” will occur on the basis of the “spleen-asthenia” constitution. On the basis of this theory, TCM JLJ granules will be developed to treat postoperative patients with CRA. Its main components include pilose asiabell roots, *Atractylodes macrocephala*, glabrous greenbrier rhizome, honeysuckle stem, *Orsythia suspensa*, *Cynanchum roseum*, etc. JLJ granules have achieved certain effects in improving postoperative symptoms of patients with CRA and preventing the recurrence of adenoma [17]. Chinese clinicians have much experience in the treatment of colorectal adenoma by traditional Chinese medicine (TCM), and during the relevant treatment process, no significant side effects or adverse reactions were observed. Lin [15] and Chen [22] conducted meta-analyses on 1103 patients from 13 studies and 1737 patients from 17 studies, respectively, and the results revealed that TCM can provide safe treatment for patients with colorectal adenoma safely without obvious adverse reactions. According to a relevant report, glabrous greenbrier rhizome in JLJ granules may cause mild adverse reactions such as vomiting, eczema, and other minor side effects [29], but within a safe dosage range, no serious adverse reactions for glabrous greenbrier rhizome or other relevant ingredients were reported. So a randomized, double-blinded, and placebo-controlled trial is described in this study to objectively and accurately evaluate the clinical efficacy of JLJ granules in the treatment of postoperative patients with CRA, observe the effect of JLJ granules on the recurrence rate of CRA, and evaluate the safety profile of JLJ granules.

However, there are also several limitations that need to be considered in this study. First of all, due to funding constraints, our follow-up period is only 12 months and we are not able to conduct a long-term follow-up observation. Moreover, the sample size included in the trial is relatively small, and the results may be biased compared with the results of multi-center and large sample size clinical trials. If high-quality evidence for the efficacy, safety, and recurrence rate of JLJ granules in the treatment of postoperative patients with CRA can be provided in this study, a large sample size, multi-center, randomized controlled, and double-blind trial will be conducted in the next step to provide a more reliable basis for JLJ granule administration in the management of postoperative CRA patients, so as to improve symptoms and avoid recurrence.

## Trial status

Recruitment for the trial is planned to start on June 2021 and last until June 2023. The trial is enrolling patients. The current protocol version is 2.0.

## Supplementary Information

**Additional file 1:** Ethics approval (Chinese Version)

**Additional file 2:** Ethics approval (English version)

**Additional file 3:** Informed consent form (Chinese version)

**Additional file 4:** Informed consent form (English version)

## Data Availability

Supporting data regarding this study protocol can be obtained as supplementary data. WLL, HW, and YZH will obtain the final trial data set and disclose the agreement. All individual participant data collected during the trial will be shared after identification.

## Abbreviations

TCM: Traditional Chinese medicine
CRAs: Colorectal adenomas
CRC: Colorectal cancer
JLJ granule: Jianpi Lishi Jiedu granule
EMR: Endoscopic mucosal resection
pEMR: Piecemeal endoscopic mucosal resection
ESD: Endoscopic submucosal dissection
