# Supplementary material for: Effects of Jianpi Lishi Jiedu granules on colorectal adenoma patients after endoscopic treatment: study protocol for a randomized, double-blinded, placebo-controlled clinical trial
Source: Trials. 2022 Apr 23;23:345. doi: 10.1186/s13063-022-06236-6 (PMC9034522; doi:10.1186/s13063-022-06236-6)
Supplement: Supplementary file 1 — Additional file 1: Ethics approval (Chinese Version) [file 13063_2022_6236_MOESM1_ESM.pdf]

南京市中西医结合医院伦理委员会

伦理审查批件

批件号：南京市中西医结合医院伦理委员会 202102

审查日期	2021 年 3 月 5 日
审查地点	南京市中西医结合医院
研究项目	健脾利湿解毒方治疗脾虚湿毒型大肠腺瘤临床疗效评价及其对 wnt/β-catenin 信号通路的影响
审查文件	伦理审查申请报告、项目临床研究方案、知情同意书
承担单位	南京市中西医结合医院,
主要研究者	刘万里
审查意见	根据《赫尔辛基宣言》和国际医学科学组织委员会颁布的《人体生物医学研究国际道德指南》的伦理原则，经本伦理委员会审查，一致同意开展“脾利湿解毒方治疗脾虚湿毒型大肠腺瘤临床疗效评价及其对 wnt/β-catenin 信号通路的影响”临床试验。<br>本批件将在医院伦理委员会备案。如果对方案实施的可行性（包括研究者的资格与经验、设备与条件等）有不同意见，请及时与本伦理委员会联系。<br>完成临床研究，请提交结题报告。<br>暂停/提前终止/完成临床研究，请及时通知伦理委员会。<br>如发生严重不良事件以及影响研究风险收益比的非预期不良事件，应及时报告本伦理委员会。<br>如临床研究方案、知情同意书的任何修改，主要研究者更换，应及时通知伦理委员会，重新审查，获得批准后执行。<br>发现影响受试者参加研究意愿的违反方案情况应及时报告。
伦理审查批件有效期	2021 年 3 月 4 日--2024 年 12 月 31 日
主任委员(副)签字	[illegible]
盖 章	南京市中西医结合医院伦理委员会
日 期	2021.3.4
