# Supplementary material for: Effects of Jianpi Lishi Jiedu granules on colorectal adenoma patients after endoscopic treatment: study protocol for a randomized, double-blinded, placebo-controlled clinical trial
Source: Trials. 2022 Apr 23;23:345. doi: 10.1186/s13063-022-06236-6 (PMC9034522; doi:10.1186/s13063-022-06236-6)
Supplement: Supplementary file 2 — Additional file 2: Ethics approval (English version) [file 13063_2022_6236_MOESM2_ESM.pdf]

Ethical Review Approval of Ethics Committee of Nanjing Integrated Traditional Chinese and Western Medicine Hospital

No：Ethics Committee of Nanjing Integrated Traditional Chinese and Western Medicine Hospital202102

Date	5 March 2021
Review location	Nanjing Integrated Traditional Chinese and Western Medicine Hospital
Invention projec	Clinical evaluation of Jianpi Lishui Jiedu Granule in the treatment of spleen deficiency and damp toxic colorectal adenoma after reatment
Documents to be reviewed	Ethical review application report、Project clinical study protocol、Informed consent
Organizer	Nanjing Integrated Traditional Chinese and Western Medicine Hospital
Main researcher	Wanli Liu
Review opinion	According to the ethical principles of the "Declaration of Helsinki" and the "International Ethical Guidelines for Human Biomedical Research" promulgated by the International Committee of Medical Science Organizations, after review by this ethics committee, it was unanimously agreed to carry out the clinical treatment of colorectal adenomas with spleen deficiency and dampness Efficacy evaluation.<br>This approval will be filed with the hospital ethics committee. If you have different opinions on the feasibility of the implementation of the scheme (including the qualifications and experience of the investigator, equipment and conditions, etc.), please contact the ethics committee in time.<br>To complete the clinical study, please submit a final report.<br>For suspension/early termination/completion of clinical research, please notify the ethics committee in time.In the event of serious adverse events and unexpected adverse events affecting the research risk-benefit ratio, they should be reported to this ethics committee in a timely manner.If the clinical research protocol or informed consent is modified, the main investigator should be changed, and the ethics committee should be notified in time, re-examined, and implemented after approval.<br>Any violation of the protocol that affects the subjects' willingness to participate in the research should be reported in time.
Valid period of Ethical Review Approval	4 March 2020—31 December 2021
Signature of Chairman	Wanli Liu
Stamp	
Date	2021.03.03
