# Supplementary material for: Effects of Jianpi Lishi Jiedu granules on colorectal adenoma patients after endoscopic treatment: study protocol for a randomized, double-blinded, placebo-controlled clinical trial
Source: Trials. 2022 Apr 23;23:345. doi: 10.1186/s13063-022-06236-6 (PMC9034522; doi:10.1186/s13063-022-06236-6)
Supplement: Supplementary file 3 — Additional file 3: Informed consent form (Chinese version) [file 13063_2022_6236_MOESM3_ESM.pdf]

知情同意书

项目名称：健脾利湿解毒方治疗脾虚湿毒型大肠腺瘤病临床疗效观察

知情同意书版本号：01，　　年　月　日（“01”为版本序号，随版本更新而依次递增）

研究机构：南京中医药大学附属南京市中西医结合医院

主要负责研究医师：刘万里 吴昊

尊敬的同志：

你好！您将被邀请参加一项临床研究。本知情同意书提供给您一些信息以帮助您决定是否参加此项临床研究。请您仔细阅读，如有任何疑问请向负责该项研究的研究者提出。

您参加本项研究是自愿的。本次研究已通过本研究机构伦理审查委员会审查。

一、本研究的研究背景与主要内容

大肠腺瘤是指起源于结直肠黏膜或黏膜下层，突出向腔内局限性生长的良性上皮隆起性病变，包括结肠与直肠腺瘤。大肠腺瘤是大肠息肉的一种，根据病理类型可将大肠息肉分为增生性息肉、炎症性息肉、腺瘤性息肉、错构瘤性息肉等几类。随着内镜及病理诊断技术的发展，大肠息肉的检出率逐年增高，大肠息肉尤以腺瘤性息肉作为大肠癌的主要癌前病变，且进行内镜下息肉切除后易复发，临床无公认特效药物可预防复发、癌变。该疾患为申报人所在科室特色优势病种，多年临床积累，凝练其中医病机特点“脾虚湿毒”，并研制健脾利湿解毒方，用于临床，疗效确切。课题组前期开展了大量临床、动物、药学研究，证实该药物安全有效，对其作用机制及药学组分进行研究，取得满意成效。我们认为该药物值得临床推广应用。

我们前期运用健脾利湿解毒颗粒治疗大肠腺瘤术后的患者取得了一定的疗效，但是其具体的的疗效尚未得到客观的验证。为进一步认识健脾利湿解毒方治疗大肠腺瘤术后的疗效、对腺瘤复发的影响及安全性，为中医药干预大肠息肉提供理论依据，因此我们在前期研究的基础上拟开展本项目工作。

二、本研究的主要目标

1、观察健脾利湿解毒方治疗大肠腺瘤性息肉的临床疗效；2、评估健脾利湿解毒方对大肠腺瘤术后患者复发的影响；3、评估健脾利湿解毒颗粒治疗大肠

腺瘤术后患者的安全性。

三、本研究的意义

大肠腺瘤性息肉临床发病率高，为大肠癌的癌前病变，临床上无法预防，切除后复发率高，影响患者生活质量，本项目的研究是课题组前期系列研究的延续和拓展，研究意义在于：

1、在大肠息肉检出率高、无特异性预防措施的情况下，我们提供了一种治疗、预防大肠腺瘤性息肉复发研究思路及有效的治疗药物。对大肠腺瘤性息肉的防治工作起到积极的推进作用。

2、进一步明确健脾利湿解毒方治疗大肠腺瘤术后患者的临床疗效及其对大肠腺瘤复发的影响，评估其安全性。

3、健脾利湿解毒方长期应用于临床，若本研究可以为健脾利湿解毒方提供一定的依据，将为新药研发提供实验室依据，为未来临床的推广应用提供基础。

本项目的研究，结合中医药的传统优势，并运用现代科技探索其科学本质，体现传承与创新并重，理论与临床相长。本项目所获得的研究成果有助于我们进一步认识利湿解毒方对大肠腺瘤的治疗机理，为中医药干预大肠腺瘤、预防复发、安全有效提供理论依据。为中医创新、发展与现代化提供科技支撑。在业内尚处于研究空白。

四、研究过程及方法

在进入本研究之前，我们会对您进行进一步的诊断和评估，如果您符合入选标准，才会建议您入组参加**本研究临床研究部分**。研究方案如下：

选取 2021 年 06 月 01 日——2023 年 06 月 01 日本院收治的确诊为大肠腺瘤性息肉的患者。采用随机的方法, 分为治疗组（健脾利湿解毒方）、对照组（随访观察）两组，治疗组给予健脾利湿解毒方口服，对照组临床随访观察，疗程为 3 个月。比较组别间、组内治疗过程中各项指标：各主要症状、次要症状、息肉复发情况、血常规、肝肾功能、心电图等；并在治疗结束后（3 个月）、6 个月、12 个月时复查肠镜。

本研究不影响您的利益。

如果您同意参与这项研究，我们将和您或您的家人进行详细沟通，向您介绍该项研究的有关情况，也请您提供与疾病有关的情况，包括发病过程、家族史、

以前就诊情况及曾经做过一些检查结果等。我们将对每位参与者进行编号，建立病历档案。

五、本研究的风险和收益

风险与不适：可能需要您向我们提供一切相关的病史，但是我们会进行绝对的保密措施。

受益：通过对您的信息资料进行研究，将为您的治疗提供必要的建议，或为疾病的研究提供有益的信息。

隐私问题：

如果您决定参加本项研究，您参加试验及在试验中的个人资料均属保密。负责研究医师及其他研究人员将使用您的医疗信息进行研究。这些信息包括您的姓名、地址、电话号码、病史及在您研究来访时得到的信息。您的医疗纪录（病历、理化检查报告等）将完整的保存在医院，医生（研究者）、专业学术委员会伦理委员会和卫生监督管理部门将被允许阅读您的医疗纪录。任何有关本项研究结果的公开报告将不会披露您的个人身份。我们将在法律允许的范围内尽一切努力保护您个人医疗资料的隐私。除本研究以外，有可能在今后的其他研究中会再次利用您的医疗纪录和检查数据。您现在也可以声明拒绝除本研究外的其他研究利用您的医疗纪录和数据信息。您可以选择不参加本项研究，或者在任何时候通知研究者后退出而不会遭到歧视或报复，您的任何医疗待遇与权益不会因此而受到影响。如某您需要其它治疗，或者您没有遵守研究计划，或者发生了与研究相关的损伤，或者有任何其它原因，可以不得到您的同意而被要求退出本项研究。

如果您因参与这项研究出现试验相关的损害，将依据法律有关规定提供相应的治疗与赔偿。

您可随时了解与本研究有关的信息资料和研究进展，如果您有与本研究有关的问题，或您在研究过程中发生了任何不适与损伤，或有关于本项研究参加者权益方面的问题您可以通过 +8613770316399（电话）与吴昊（负责人）联系。

知情同意书

我已经阅读了本知情同意书。

我有机会提问而且所有问题均已得到解答。

我理解参加本项研究是自愿的。

我可以选择不参加本项研究，或者在任何时候通知研究者后退出而不会遭到歧视或报复，我的任何医疗待遇与权益不会因此而受到影响。

如果我需要其它治疗，或者我没有遵守研究计划，或者发生了与研究相关的损伤或者有任何其它原因，研究医师可以终止我继续参与本项研究。

如果我参与这项研究出现试验相关的损害，责任方将依据法律有关规定提供相应的治疗与赔偿。

我将收到一份签过字的“知情同意书”副本。

受试者姓名：________________________

受试者签名：_________________________

日期：________年________月________日

我已准确地将这份文件告知受试者，他/她准确地阅读了这份知情同意书，并证明该受试者有机会提出问题。我证明他/她是自愿同意的。

研究者姓名：________________________

研究者签名：_________________________

日期：________年________月________日

（注：如果受试者不识字时尚需见证人签名，如果受试者无行为能力时则需代理人签名）
