# Supplementary material for: Effects of Jianpi Lishi Jiedu granules on colorectal adenoma patients after endoscopic treatment: study protocol for a randomized, double-blinded, placebo-controlled clinical trial
Source: Trials. 2022 Apr 23;23:345. doi: 10.1186/s13063-022-06236-6 (PMC9034522; doi:10.1186/s13063-022-06236-6)
Supplement: Supplementary file 4 — Additional file 4: Informed consent form (English version) [file 13063_2022_6236_MOESM4_ESM.pdf]

Informed Consent Form

Project Name: Clinical Observation of Jianpi Lishi Jiedu granule in Treating Colorectal Adenoma of Spleen Deficiency and Dampness Toxicity Type

Version No.2 of ICF: 01, 14 March 2021 ("01" is the serial number of the version, increasing in turn with the update of the version)

Research Institution: Nanjing Integrated Traditional Chinese and Western Medicine Hospital Affiliated to Nanjing University of Traditional Chinese Medicine

Physician: Wanli Liu and Hao Wu

Dear Comrades,

You will be invited to participate in a clinical study. This ICF provides you with some information to help you decide whether to participate in this clinical study. Please read it carefully. If you have any questions, please ask the researcher in charge of the study.

Your participation is voluntary. This research has been reviewed by the Ethics Review Committee of this research institution.

I. Background and Contents

Colorectal adenoma refers to benign epithelial protruding lesions originating from colorectal mucosa or submucosa and protruding into the lumen to grow locally, including colorectal adenoma. Colorectal adenoma is a kind of colorectal polyps. According to pathological types, colorectal polyps can be divided into hyperplastic polyps, inflammatory polyps, adenomatous polyps, hamartomatous polyps and so on. With the development of endoscopic and pathological diagnosis technology, the detection rate of colorectal polyps is increasing year by year. Colorectal polyps, especially adenomatous polyps, are the main precancerous lesions of colorectal cancer, and are easy to recur after endoscopic polypectomy. There is no clinically recognized specific medicine to prevent recurrence and canceration. This disease is a characteristic and dominant disease in the department where the applicant is located. It has

accumulated clinically for many years, condensed the pathogenesis characteristic of traditional Chinese medicine, "spleen deficiency and dampness toxin", and developed a prescription for invigorating spleen, promoting diuresis and detoxifying, which is used clinically with definite curative effect. The research group carried out a large number of clinical, animal and pharmaceutical studies in the early stage, which proved that the medicine was safe and effective. The mechanism of action and pharmaceutical components were studied and satisfactory results were achieved. We believe that this medicine is worthy of clinical application.

We used Jianpi Lishi Jiedu Granule to treat postoperative patients with colorectal adenoma in the early stage and achieved certain curative effect, but its specific curative effect has not been objectively verified. In order to further understand the curative effect of Jianpi Lishi Jiedu granule on postoperative colorectal adenoma, the influence on recurrence of adenoma and its safety, and to provide theoretical basis for traditional Chinese medicine intervention on colorectal polyps, we plan this project on the basis of previous research.

II. Main Objectives

1. To observe the clinical effect of Jianpi Lishi Jiedu granule on colorectal adenomatous polyps; 2. To evaluate the effect of Jianpi Lishi Jiedu granule on recurrence of postoperative patients with colorectal adenoma; 3. To evaluate the safety of Jianpi Lishi Jiedu Granule in treating postoperative patients with colorectal adenoma.

III. Significance

Colorectal adenomatous polyp has a high clinical incidence rate, is a precancerous lesion of colorectal cancer, cannot be prevented clinically, has a high recurrence rate after resection, and affects the quality of life of patients. The research of this project is the continuation and expansion of the previous series of studies of the research group. The research significance lies in:

1. Under the condition of high detection rate of colorectal polyps and no specific preventive measures, we provide a research idea and effective

therapeutic medicines for treatment and prevention of recurrence of colorectal adenomatous polyps. It plays a positive role in the prevention and treatment of colorectal adenomatous polyps.

2. Further clarify the clinical efficacy of Jianpi Lishi Jiedu granule in treating postoperative patients with colorectal adenoma and its influence on recurrence of colorectal adenoma, and evaluate its safety.

3. Jianpi Lishi Jiedu granule has been used clinically for a long time. If this study can provide a certain basis for Jianpi Lishi Jiedu granule, it will provide laboratory basis for new medicine research and development, and provide a basis for future clinical popularization and application.

The research of this project combines the traditional advantages of traditional Chinese medicine and uses modern science and technology to explore its scientific essence, which embodies equal emphasis on inheritance and innovation, and mutual benefit between theory and clinic. The research results obtained in this project are helpful for us to further understand the therapeutic mechanism of Lishi Jiedu Recipe on colorectal adenoma, and provide theoretical basis for Chinese medicine to intervene colorectal adenoma, prevent recurrence, and be safe and effective, as well as scientific and technological support for the innovation, development and modernization of traditional Chinese medicine. It is still in the research blank in the industry.

IV. Process and Methods

Before entering this study, we will further diagnose and evaluate you. If you meet the inclusion criteria, you will be recommended to participate in the clinical research part of this study. The research programme is as follows:

From June 1st, 2021 to June 1st, 2023, patients diagnosed as adenomatous polyps of large intestine were selected. They were randomly divided into treatment group (Jianpi Lishi Jiedu granule) and control group (follow-up observation). The treatment group was given Jianpi Lishi Jiedu granule orally, while the control group was followed up for 3 months. Compare the indexes during treatment between groups and within groups: main symptoms,

secondary symptoms, polyp recurrence, blood routine, liver and kidney function, electrocardiogram, etc. After treatment (3 months), 6 months and 12 months, colonoscopy was reexamined.

This study does not affect your interests.

If you agree to participate in this study, we will communicate with you or your family in detail, introduce you to the relevant information of this study, and ask you to provide information related to the disease, including the onset process, family history, previous visits and some examination results, etc. We will number each participant and establish a medical record.

V. Risks and Benefits

Risks and discomfort: You may need to provide us with all relevant medical history, but we will take absolute confidentiality measures.

Benefits: By studying your information, it will provide necessary suggestions for your treatment or provide useful information for disease research.

Privacy:

If you decide to participate in this study, your participation in the experiment and your personal data during the experiment will be kept confidential. Doctors and other researchers in charge of research will use your medical information for research. This information includes your name, address, telephone number, medical history and information you got during your research visit. Your medical records (medical records, physical and chemical examination reports, etc.) will be completely kept in the hospital, and doctors (researchers), ethics committees of professional academic committees and health supervision and management departments will be allowed to read your medical records. Any public report on the results of this research will not disclose your personal identity. We will make every effort to protect the privacy of your personal medical data within the scope permitted by law. In addition to this study, it is possible to reuse your medical records and examination data in other future studies. You can now also declare that you refuse to use your medical records

and data for studies other than this one. You can choose not to participate in this study, or withdraw after notifying the researcher at any time without discrimination or retaliation, and your medical treatment and rights will not be affected. If you need other treatment, or you have not complied with the research plan, or have an injury related to the research, or for any other reason, you can be asked to withdraw from this research without your consent.

If you suffer from test-related damage due to your participation in this study, you will provide corresponding treatment and compensation according to the relevant provisions of the law.

You can know the information and research progress related to this study at any time. If you have any problems related to this study, or if you have any discomfort or injury during the study, or if you have any problems about the rights and interests of participants in this study, you can contact Hao Wu (responsible person) at +8613770316399 (telephone).

Informed Consent Form

I have read this ICF.

I had the opportunity to ask questions and all the questions have been answered.

I understand that participation in this study is voluntary.

I can choose not to participate in this study, or withdraw after notifying the researcher at any time without discrimination or retaliation, and any of my medical treatment and rights will not be affected.

If I need other treatment, or I dont follow the research plan, or I have an injury related to the research or for any other reason, the research physician can terminate my participation in this research.

If I participate in this study and suffer damage related to the test, the responsible party will provide corresponding treatment and compensation according to the relevant provisions of the law.

I will receive a signed copy of the Informed Consent Form.

Name of subject: ________________________

Subject signature: ________________________

Date: MM/DD/YY

I have accurately informed the subject of this document, and he/she has accurately read this ICF and proved that the subject has the opportunity to ask questions. I certify that he/she gave his/her consent voluntarily.

Name of researcher: ________________________

Signature of researcher: ________________________

Date: MM/DD/YY

(Note: If the subject is illiterate, a witnesss signature is required, and if the subject is incapacitated, an agents signature is required)
